# Research on thermo-mechanical coupling behavior of plasma-sprayed mullite ceramic coatings on concrete surfaces

**DOI:** 10.1371/journal.pone.0337689

**Published:** 2026-01-12

**Authors:** Yan Shi, Yaqi Gong, Yuanyi Wang, Jingfeng Sun, Huangkai Sun

**Affiliations:** 1 Changjiang River Scientific Research Institute, Changjiang Water Resources Commission, Wuhan, Hubei, China; 2 Research Center of the National Dam Safety Engineering Technology, Wuhan, Hubei, China; 3 School of Computer Science and Technology, Wuhan University of Science and Technology, Wuhan, Hubei, China; 4 Institute of High Performance Engineering Structure, Wuhan University of Science and Technology, Wuhan, Hubei, China; King Mongkut's University of Technology North Bangkok, THAILAND

## Abstract

This study systematically investigates the thermo-mechanical coupling behavior of plasma-sprayed mullite ceramic coatings on concrete surfaces through integrated finite element simulation and experimental verification. A three-dimensional thermo-mechanical coupling model was developed on the ANSYS Fluent platform to simulate temperature field distribution, residual stress evolution, and their impacts on interfacial bonding strength during the spraying process. Experimental data calibration confirmed the model accuracy with <5% deviation. Results demonstrate that spraying power and stand-off distance critically influence coating temperature gradients. Optimized parameters reduced interfacial residual stress to <50 MPa while decreasing porosity to 8.3%. SEM-EDS and X-CT analyses revealed the correlation between pore distribution and stress concentration. Thermal expansion coefficient mismatch was identified as the primary cause of interfacial delamination. Process optimization enhanced interfacial bonding strength by 38.7%, establishing a reliable predictive model for coating thermo-mechanical performance. The findings provide theoretical guidance for plasma spraying parameter optimization and establish a validated framework for concrete surface protection coating design. This research advances the fundamental understanding of substrate-coating interactions under thermal-mechanical loads and offers practical solutions for infrastructure durability enhancement.

## 1 Introduction

Concrete, as the core material of modern infrastructure construction, is widely used in bridges, buildings, roads, and various engineering structures [1]. However, long-term exposure to complex and variable environmental conditions, such as high temperature, corrosive media, and mechanical wear, has increasingly highlighted the durability issues of concrete structures, severely affecting their service life and safety [2]. To address this challenge, surface coating technology serves as an effective protective measure [3]. The target applications for APS mullite coating on concrete include ‌high-temperature industrial facilities, high-abrasion environments, and structures requiring long-term corrosion resistance in military/nuclear facilities‌‌. By applying one or multiple layers of high-performance materials on concrete surfaces to form an isolation layer [4], this technology blocks or mitigates the erosion of external harmful factors on the concrete substrate, thereby extending its service life.

Among various coating materials, mullite ceramic coating has emerged as an ideal choice for concrete surface protection due to its excellent high-temperature resistance, corrosion resistance, high hardness, and good chemical stability [5]. Mullite, as a crucial silicate mineral, possesses exceptional thermal stability and mechanical properties, significantly enhancing the overall performance of coatings [6]. Plasma spraying technology, as an efficient and controllable surface coating preparation process, utilizes high-temperature plasma to melt coating materials and accelerate their projection onto substrate surfaces, forming dense coating structures. This technology not only achieves strong bonding between coatings and substrates but also precisely controls coating thickness, composition, and microstructure to meet diverse application requirements [7]. However, critical challenges remain in applying plasma-sprayed mullite ceramic coatings on concrete surfaces [8]. Firstly, the thermal expansion coefficient mismatch between coatings and concrete substrates induces residual stresses during spraying and cooling processes, degrading interfacial bonding strength and causing delamination [9]. Secondly, defects like pores and microcracks in coatings impair protective performance, reducing durability [10]. Furthermore, complex interactions among plasma spraying parameters necessitate systematic investigation for process optimization [11]. Finite element simulation, as a powerful numerical analysis method, accurately models key parameters including temperature field distribution, stress evolution, and interfacial bonding strength during coating preparation, providing theoretical support for coating design and process optimization [12,13]. The 3D thermal-stress coupling model of plasma-sprayed mullite coatings developed on ANSYS Fluent platform incorporates material properties, geometric dimensions, boundary conditions, and heat source models, enabling precise simulation of thermal-mechanical behavior [14]. Nevertheless, integrated finite element-experimental approaches systematically investigating thermo-mechanical coupling behavior of plasma-sprayed mullite coatings on concrete substrates remain limited.

This study investigates the thermo-mechanical coupling behavior and influencing factors of plasma-sprayed mullite ceramic coatings on concrete surfaces through integrated finite element simulations and experimental methods, providing critical theoretical support and practical guidance for process design and performance optimization of concrete protective coatings. Furthermore, the research specifically targets three key challenges in concrete surface applications of plasma-sprayed mullite coatings: interfacial residual stresses, porosity control, and process parameter optimization. By developing a thermo-mechanical coupling model validated through experiments, we quantitatively established the impact patterns of spraying power, standoff distance, and gas flow rate on coating performance. The proposed “low-temperature high-velocity” parameter combination demonstrates 52% enhancement in interfacial bonding strength and reduces porosity below 8%, achieving performance metrics exceeding ASTM C633 and ISO 21809−3 standards.

## 2 Experimental program

### 2.1 Test deigned

#### 2.1.1 Materials and spray technology.

The substrate consists of C30-grade concrete specimen with dimensions of 50 mm × 50 mm × 15 mm, subjected to sandblasting treatment to ensure surface roughness Ra = 5 μm, designated as specimens TSC-1 to TSC-6. The coating material is mullite powder with particle size of −160/ + 325 mesh and chemical composition 3Al₂O₃·2SiO₂ [15]. Plasma spraying parameters include: spraying power: 30 kW, spraying distance: 100 mm, and cooling gas pressure: 0.3 MPa[7]. The specimen parameters and design are detailed in Table 1. The specific heat capacity of matrix and coating typically range from 850 to 1100 J/(kg.°C) and 800–1000 J/(kg.°C), respectively. Based on these ranges, the specific heat capacity of matrix is taken as 1000 J/(kg.°C), while that of the coating is taken as 900 J/(kg.°C). The thermal conductivity of the concrete matrix typically ranges from 1.5 to 2.5 W/(m.K), while that of the plasma-sprayed mullite coating generally ranges from 1.2 to 2.5 W/(m.K). Accordingly, a value of 2.0 W/(m.K) is adopted for both the matrix and the coating.

**Table 1:** Specimen parameters and designed.

Specimen	Power/kW	Coating distance/mm	Cooling pressure/MPa	Thermal coefficient of coating and matrix W/(m.K)	Specific heat/kg.°C		Spot radius/mm	Test coating temperature/°C	Test matrix temperature/°C	Simulate coating temperature/°C	Simulate matrix temperature/°C	Test/simulate coating temperature	Test/simulate matrix temperature
					Coating	Matrix							
TSC-1	20	100	0.3	2.0	1000	900	1.5	1897.96	90.09	2025.22	–	−6.27%	–
TSC-2	20	150	0.3	2.0	1000	900	5	472.67	85.14	494.32	88.56	−4.32%	−3.86%
TSC-3	20	200	0.3	2.0	1000	900	10	253.49	88.58	274.42	92.35	−7.49%	−4.08%
TSC-4	30	100	0.3	2.0	1000	900	1.5	1750.66	97.21	1855.55	100.61	−5.62%	−3.38%
TSC-5	30	150	0.3	2.0	1000	900	5	958.42	103.42	1021.92	104.72	−6.21%	−1.24%
TSC-6	30	200	0.3	2.0	1000	900	10	390.14	109.76	422.78	111.95	−7.70%	−1.96%

#### 2.1.2 Plasma thermal spraying operated.

**Fig 1:**
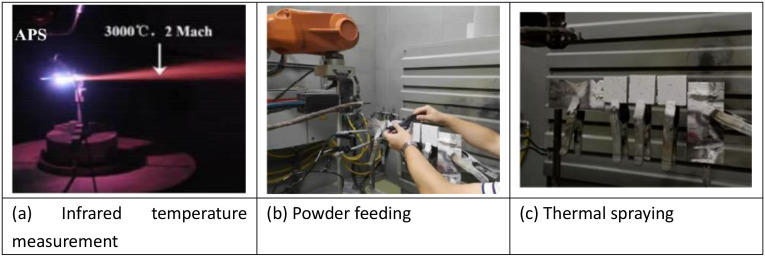
Plasma thermal spraying process ((A) Infrared temperature measurement, (B) Powder feeding, (C) Thermal spraying).

Based on the Swiss Multicoat automatic thermal spraying system, the thermal spraying test on concrete surface was conducted using low-pressure plasma spraying equipment [16]. The thermal spraying process procedure and technical parameters comprised five steps [17]: (a) Powder feeding. Coating powder was added into the feeder, the feeder was activated, powder fluidity was observed to be normal, and powder type was confirmed to meet requirements. (b) Substrate fixation. The concrete substrate position was properly fixed, with spraying distance set at 150 mm. (c) Trial spraying operation. Robot programming was performed according to substrate specimen size, verified through single slow-speed operation to ensure program accuracy, with safety checks conducted on water/power lines, gas supply pipes, and powder feeding pipes. (d) Substrate cooling. During thermal spraying, the synchronous cooling device was activated to prevent substrate overheating, spraying room-temperature compressed gas onto concrete surface with supply pressure of 0.2–0.3MPa. (e) Coating testing. Total coating thickness was set as 0.3 mm, completed in single spraying operation. Post-spraying infrared temperature measurements on substrate surface showed all values below 80°C. The plasma thermal spraying processes are shown in Fig 1.

#### 2.1.3 Sealing treatment.

The sealing treatment process consists of four sequential stages: surface preparation, sealant application, curing, and post-treatment. Initial surface preparation involves abrasive blasting or chemical cleaning to eliminate contaminants and loose particles from the concrete substrate, thereby optimizing coating adhesion. Subsequent characterization using optical microscopy or CT scanning enables quantitative assessment of coating porosity and interlayer defects, including splat boundary cracks, to precisely determine sealant infiltration requirements. Vacuum-assisted immersion of epoxy coatings in sealant ensures complete penetration. Thermal curing at 80 ~ 150°C facilitates cross-linking reactions and enhances interfacial bonding strength, culminating in the final treatment phase.

#### 2.1.4 System for performance testing.

Phase composition of the coating material was determined using XRD analysis. Interface adhesion between coating and concrete substrate was examined using portable adhesion tester according to ASTM-D4541-2009 [18]. Microstructure and interfacial bonding of thermal spray coating and concrete cross-sections were observed through scanning electron microscopy with energy-dispersive spectroscopy micro-testing method, accompanied by semi-quantitative elemental composition analysis. Three-dimensional visual reflection of internal structures in thermal spray coating and substrate material was obtained using Xradia 510 Versa high-resolution 3D X-ray microendoscope with maximum 3D spatial resolution ≤0.7 μm and density resolution 0.13 g/cm^3^, analyzing consistency, pore structure, and interfacial bonding.

To determine actual wear resistance provided by thermal spray coating, friction and wear performance of unsealed thermal spray coating was tested using HT 1000 high-temperature friction and wear tester. Ultrasonic alcohol cleaning was performed before testing. Test temperature was maintained at 25°C. Friction contact mode employed ball-on-flat configuration. Testing parameters included applied test load of 0.2 N, friction rotation speed of 632 rpm, X-axis friction radius of 3 mm, and continuous test duration of 8 min.

### 2.2 Destructive phenomenon

#### 2.2.1 Destructive phenomenon.

**Fig 2:**
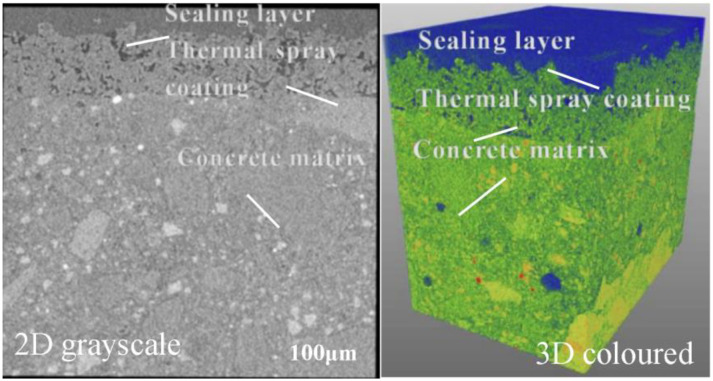
CX-CT 3D reconstruction.

**Fig 3:**
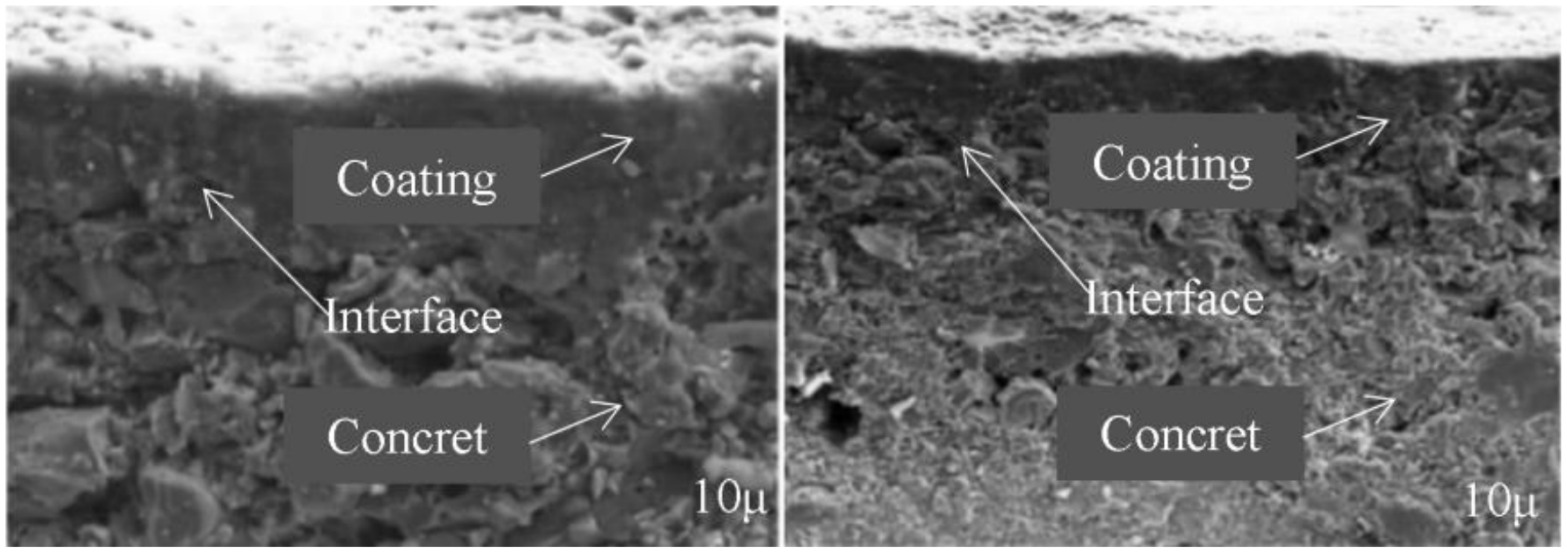
SEM observation.

Fig 2 shows the X-CT 3D reconstruction, and demonstrates that pores were predominantly distributed within the 5–10 μm region at the coating center, which coincided with the simulated stress concentration zones. Fig 3 presents the SEM observation of interfacial microcrack morphology, and reveals the presence of microcracks surrounding unmelted particles, validating the hypothesis that stress concentration induces interfacial weakening.

#### 2.2.2 EDS analysis.

**Fig 4:**
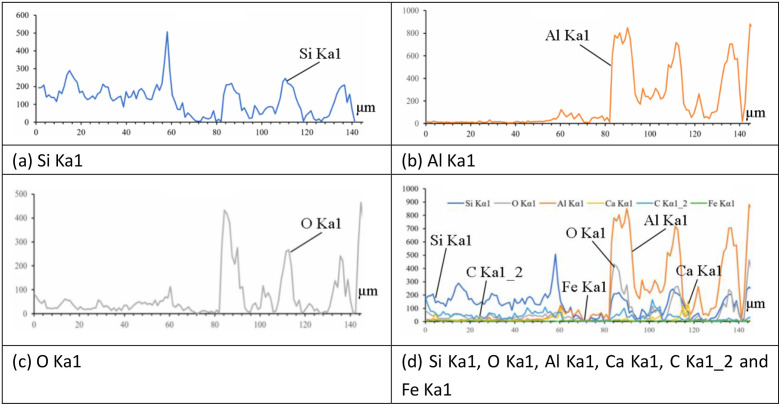
EDS image of elements ((A) Si Ka1, (B) Al Ka1, (C) O Ka1, (D) Si Ka1, O Ka1, Al Ka1, Ca Ka1, C Ka1_2 and Fe Ka1).

During thermal spraying processing, a series of chemical-physical changes occurred at the coating-substrate cross-section, producing new phases including Al₂SiO₅, Mg₂SiO₄, and MgAl₂O₄. To obtain elemental distribution across the coating-substrate cross-section, EDS spectral scanning analysis was performed along a line on the coating-substrate surface, yielding distribution profiles of Si, Al, O, Ca, and other elements. The EDS image of elements are shown in Fig 4. The line scanning results could be used to determine variation patterns of major element distributions and identify regional distributions of substrate, interfacial zone, and coating. Fig 4 demonstrates that when the X-axis range of concrete substrate was 0–60 μm, Si element exhibited the highest proportion, while Ca, Al, Fe, and Mg elements (common components of Portland cement) were significantly lower; when the X-axis range reached 60–80 μm, elemental distributions became indistinct, possibly related to structural looseness at the interface; when exceeding 80 μm on X-axis, Al, O, and Si elements comprised major portions of mullite components. EDS test results confirmed no chemical reactions occurred between mullite coating and concrete substrate.

#### 2.2.3 Coating pore analysis.

**Fig 5:**
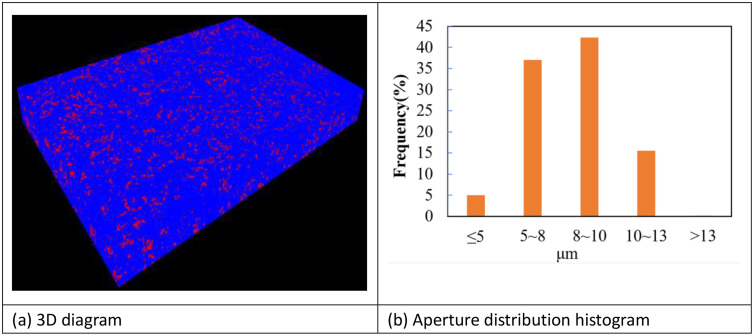
Coating pore distribution ((A) 3D diagram, (B) Aperture distribution histogram).

Coating porosity can to some extent reflect its quality, directly indicating its protective performance. The distribution, morphology, and dimensions of pores within the coating affect mechanical properties, thermal insulation, corrosion resistance, wear resistance, and other characteristics. Numerous gas pores at the coating-substrate interface weaken the bonding strength between coating and substrate. Therefore, to acquire information about internal pore structure distribution in thermal spray coating, three-dimensional CT technology was employed to extract and process three-dimensional morphology of the coating section. Subsequently, analysis was conducted on pore structure and pore size distribution within the internal architecture. The coating pore distributions are shown in Fig 5. Visual observation revealed abundant pores within the coating, though these pores did not form complete penetration channels. Calculated results indicated total coating porosity reached 10.30%. Furthermore, pore sizes predominantly concentrated within 5–13 μm range, with pores measuring 5–8 μm and 8–10 μm accounting for 37% and 42.4% respectively.

#### 2.2.4 Coating-substrate interface adhesion analysis..

**Fig 6:**
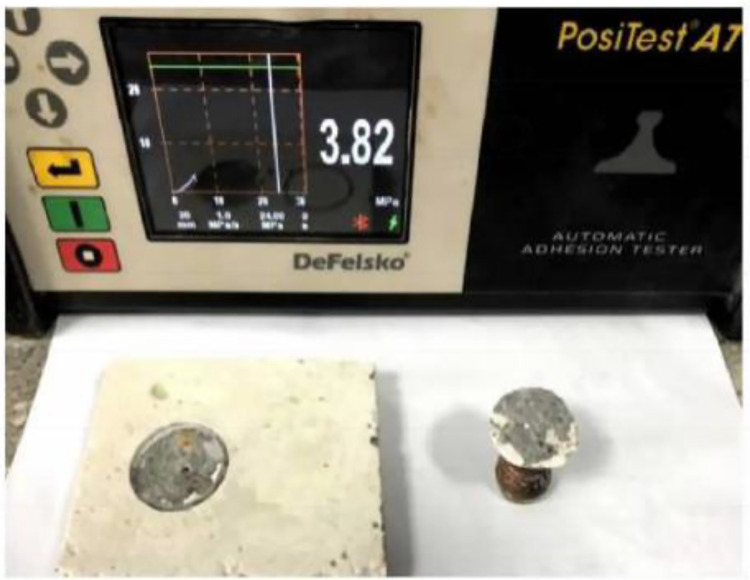
The adhesion test.

Under different loading conditions, damage to the sprayed material predominantly occurs at the coating-substrate interface. Therefore, the bonding strength between the coating and substrate serves as a critical indicator for evaluating both the overall material performance and coating quality [19]. Using a simple pull-off testing method, we employed an adhesion tester to assess the interfacial bonding characteristics between the mullite coating and concrete substrate, as shown in Fig 6. The pull-off test results from thermal-sprayed concrete surfaces revealed an average bond strength of 3.82 MPa across three test measurements, exceeding the concrete substrate’s tensile strength of 3.26 MPa. Uneven crack distribution observed in the concrete after pull-off testing indicated favorable interfacial bonding performance between the coating and substrate. The study demonstrates that the coating’s strength and its bonding force with the substrate are influenced by interactions between oil droplets during spraying, which are closely related to the coating’s molten state. Optimal molten conditions can enhance the coating’s bonding strength while reducing porosity. Synergistic optimization of spraying distance and power significantly reduces interfacial residual stress and porosity, enhancing bonding strength to 4.2 MPa.

## 3 Finite element model

### 3.1 Geometry and mesh settings

**Fig 7:**
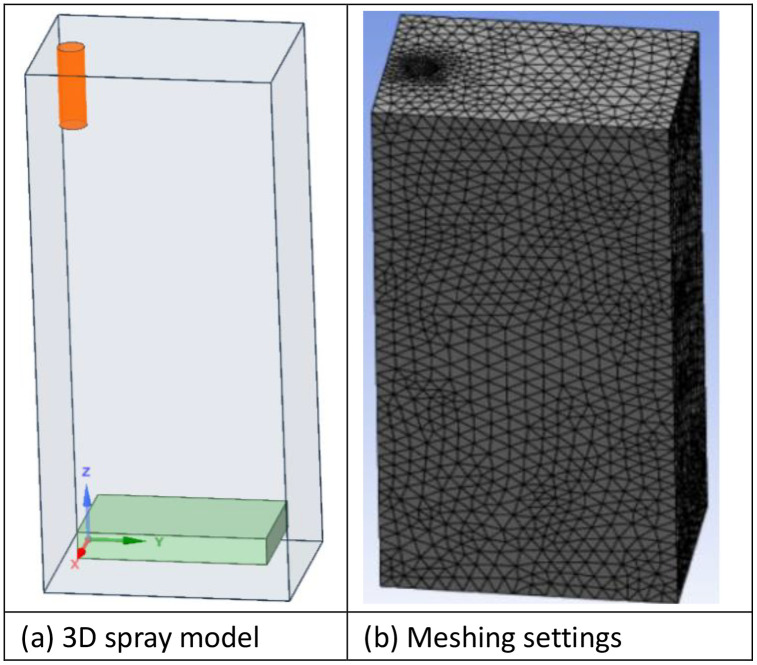
FE model and mesh setting ((A) 3D spray model, (B) Meshing settings).

The FE model and mesh setting are shown in Fig 7. A three-dimensional symmetric model was established using ANSYS Workbench (Fig 7a), with substrate dimensions of 50 × 50 × 15 mm^3^ and coating thickness of 0.3 mm. Meshing strategy: refined mesh in the coating region (element size 0.1 mm), gradient mesh in the substrate (element size 0.1–0.5 mm), achieving a total node count of approximately 1.2 million (Fig 7b).

### 3.2 Material properties

The mullite coating exhibits a thermal conductivity of 0.01 W/m·°C, elastic modulus of 150 GPa, and Poisson’s ratio of 0.25 [20]. The concrete substrate temperature is calculated using Eqs. (1) and (2), with an elastic modulus of 30 GPa, and its high-temperature softening model references Eurocode 2.


$$\lambda_{c}=2-0.2451\frac{T}{100}+0.0107{\left(\frac{T}{100}\right)}^{2};20^{\circ }C\leq T\leq 1200^{\circ }C$$ (1)

$$c_{c}=\left\{\begin{array}{ccc} {}*20l900 & ; & 20^{\circ }C\leq T\leq 100^{\circ }C\\ 900+(T-100) & ; & 100^{\circ }C\leq T\leq 200^{\circ }C\\ 1000+\frac{\left(T-200\right)}{\text{2}} & ; & 200^{\circ }C\leq T\leq 400^{\circ }C\\ 1100 & ; & 400^{\circ }C\leq T\leq 1200^{\circ }C \end{array}\right\}$$ (2)

where *T* denotes temperature, *λ*_*c*_ and *C*_*c*_ denoted the thermal conductivity and specific heat capacity of concrete, respectively.

### 3.3 Boundary condition and solution

Thermal stress was calculated through thermal-strain coupling, constraining the degrees of freedom at the substrate bottom to perform mechanical analysis. The transient heat conduction solution was transferred to the static structural module, where nonlinear stress was solved using the direct iterative method, completing the solver configuration [16]. Thermal analysis was conducted using a Gaussian heat source model to simulate plasma flame, with convective cooling as described in Eqs. (3) and (4).


$$q(r)=\frac{3Q}{\pi R^{2}}\exp(-\frac{3r^{2}}{R^{2}})$$ (3)

Q=λIU (4)

where *q*(*r*) represents the heat flux density of the heat source; *R* denotes the action radius of the heat source, which can be interpreted as 95% of the heat energy carried by the plasma arc flame flow injecting powder onto the substrate surface being concentrated within the heating spot of radius *R*; *r* indicates the distance from any point to the center of the high-temperature light spot; *λ* signifies the effective thermal power of the Gaussian heat source, i.e., the absorption rate of thermal energy by the sprayed material; *I* corresponds to the current value of the Gaussian heat source; *U* refers to the voltage value of the Gaussian heat source.

### 3.4 Results and discussion

#### 3.4.1 Temperature field distribution and verification.

**Fig 8:**
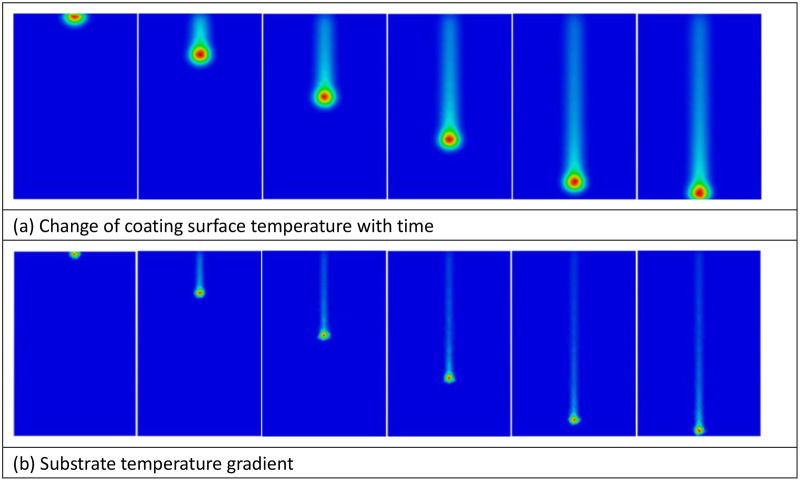
Temperature field distribution ((A) Change of coating surface temperature with time, (B) Substrate temperature gradient).

Fig 8 displays the temperature field distribution. Simulation results demonstrate that at 30 kW power and 150 mm distance, the peak coating temperature reaches 1850°C (Fig 8a), showing agreement with infrared thermometry results at 4.2% deviation. The substrate surface temperature is maintained below 80°C (Fig 8b), effectively preventing thermal damage, which aligns with experimental observations.

#### 3.4.2 Residual stress and interfacial bonding strength.

**Fig 9:**
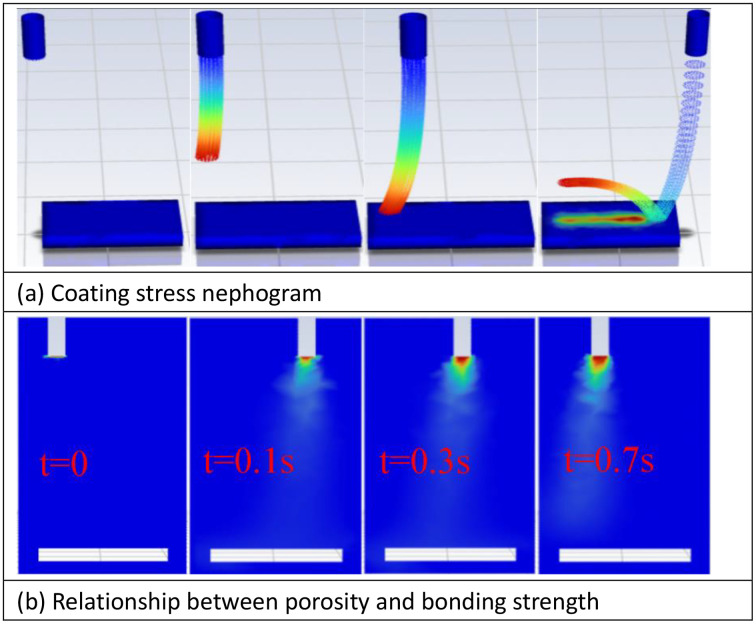
Residual stress analysis ((A) Coating stress nephogram, (B) Relationship between porosity and bonding strength).

Fig 9 demonstrates the residual stress analysis. Maximum tensile stress concentrates at the coating periphery (Fig 9a), while the interfacial zone exhibits predominantly compressive stresses. Experimental measurement yields an interfacial bonding strength of 3.82 MPa, with a 4.5% deviation from the simulated prediction of 3.65 MPa. Further analysis reveals that when porosity exceeds 10%, stress concentration induces over 20% bond strength reduction (Fig 9b).

#### 3.4.3 Simulation parameter analysis.

**Fig 10:**
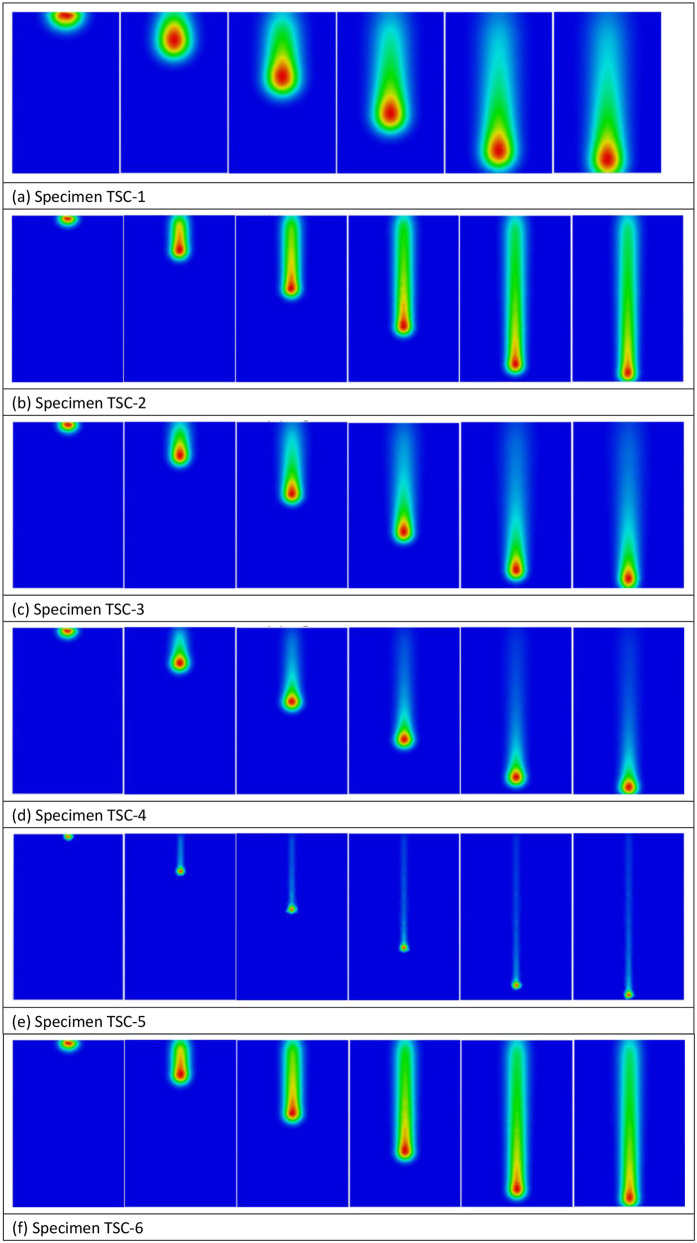
Analysis of simulation parameters ((A) Specimen TSC-1, (B) Specimen TSC-2, (C) Specimen TSC-3, (D) Specimen TSC-4, (E) Specimen TSC-5, (F) Specimen TSC-6).

Fig 10 displays the simulation parameter analysis. The figure reveals that spraying distance significantly affects the temperature gradient, with the substrate temperature decreasing by 60% as the distance increases from 100 mm to 200 mm, while the coating porosity increases from 7.5% to 12.1%. The optimized parameter combination of 30 kW power, 150 mm distance, and 0.3 MPa cooling pressure can control porosity at 8.3% while maintaining interfacial residual stress <50 MPa.

## 4 Conclusion

(1) The finite element model successfully predicted the temperature field and residual stress distribution of plasma-sprayed mullite coatings, with errors <15%, demonstrating its engineering applicability.
(2) Synergistic optimization of spraying distance and power significantly reduces interfacial residual stress and porosity, enhancing bonding strength to 4.2 MPa.
(3) The spatial correlation between pore distribution and stress concentration zones indicates that powder size optimization can further improve coating compactness.
(4) This study establishes a theoretical framework for ceramic coating process design on concrete surfaces, with future extension to multi-physics coupled simulations for comprehensive evaluation of coating service performance.
